# N-Glycosylation Regulates Pannexin 2 Localization but Is Not Required for Interacting with Pannexin 1

**DOI:** 10.3390/ijms19071837

**Published:** 2018-06-22

**Authors:** Rafael E. Sanchez-Pupo, Danielle Johnston, Silvia Penuela

**Affiliations:** Department of Anatomy and Cell Biology, Schulich School of Medicine & Dentistry, University of Western Ontario, London, ON N6A5C1, Canada; rsnchezp@uwo.ca (R.E.S.-P.); Danielle.Johnston@schulich.uwo.ca (D.J.)

**Keywords:** pannexin, Panx1, Panx2, post-translational modification, traffic, N-glycosylation, channels, subcellular localization

## Abstract

Pannexins (Panx1, 2, 3) are channel-forming glycoproteins expressed in mammalian tissues. We previously reported that N-glycosylation acts as a regulator of the localization and intermixing of Panx1 and Panx3, but its effects on Panx2 are currently unknown. Panx1 and Panx2 intermixing can regulate channel properties, and both pannexins have been implicated in neuronal cell death after ischemia. Our objectives were to validate the predicted N-glycosylation site of Panx2 and to study the effects of Panx2 glycosylation on localization and its capacity to interact with Panx1. We used site-directed mutagenesis, enzymatic de-glycosylation, cell-surface biotinylation, co-immunoprecipitation, and confocal microscopy. Our results showed that N86 is the only N-glycosylation site of Panx2. Panx2 and the N86Q mutant are predominantly localized to the endoplasmic reticulum (ER) and cis-Golgi matrix with limited cell surface localization was seen only in the presence of Panx1. The Panx2 N86Q mutant is glycosylation-deficient and tends to aggregate in the ER reducing its cell surface trafficking but it can still interact with Panx1. Our study indicates that N-glycosylation may be important for folding and trafficking of Panx2. We found that the un-glycosylated forms of Panx1 and 2 can readily interact, regulating their localization and potentially their channel function in cells where they are co-expressed.

## 1. Introduction

Pannexins (Panx1, Panx2, and Panx3) are membrane-spanning glycoproteins capable of forming large pore channels that allow the passage of ions and macromolecules involved in paracrine and autocrine signaling [1,2]. Panx1 is the most widely expressed pannexin and the most studied, with evidence supporting that its channels act as ATP and Ca^2+^ conduits [3,4,5,6] and are implicated in critical cellular processes such as cell death after brain ischemia [7] and inflammation [8]. On the other hand, Panx2 is the largest member of the family and its expression was thought to be restricted to the central nervous system (CNS). Recently, it has been reported that Panx2 can also be expressed in other tissues such as skin, kidney and liver [9,10], while Panx3 is predominantly expressed in skin, cartilage and bone [4,5,11,12,13]. In contrast to the hexameric type of channels formed by Panx1, it has been suggested that Panx2 can form octameric or heptameric channels [14] and it is still unclear whether its channel function would be similar to the other family members. Interestingly, Panx1 and Panx2 expression have been found to overlap in adult rodent brains although they are inversely regulated throughout development, with Panx1 being more abundant in neonatal and young tissues, whereas Panx2 is more abundant in the adult [15,16,17,18]. Under ischemic conditions, both Panx1 and Panx2 are expressed in the brain and their overlapping channel functions contribute to neurodegeneration. In fact, the deletion of Panx1 and Panx2 in a double knockout mouse model was necessary to observe a reduction in cell death after ischemia, perhaps due to their redundant and/or complementary functions [19].

N-glycosylation is a posttranslational modification that occurs in the endoplasmic reticulum (ER) and is recognized to have profound effects on protein folding and trafficking of membrane-bound proteins [20]. The prediction of putative N-linked glycosylation sites for pannexins has been done in the past, and it has been demonstrated that Panx1 and Panx3 have sites for N-linked glycosylation at Asn (N) 254 and N71, respectively. These studies comprised further characterization using enzymatic digestion with endoglycosidases that confirmed that all three members of the pannexin family are differentially N-glycosylated but not O-glycosylated [11,21]. However, for Panx2 the predicted site of N-glycosylation at residue N86 remains to be validated [22].

Unlike other pannexins, Panx2 is only modified to a high-mannose glycosylation species (termed as Gly-1) and presents a predominantly intracellular localization that has been associated with this lower level of N-glycosylation [22,23,24,25,26]. Previous evidence supports the concept that complex glycoprotein species (Gly-2) (further processed at Golgi) present in Panx1 and Panx3 traffic readily to the cell surface [22]. However, our group and others have stated that under certain circumstances Panx2 can also translocate to the plasma membrane [14,17,22], but it is still unclear whether glycosylation plays a role in regulating Panx2 trafficking.

Panx2 has been shown to interact with Panx1, and when co-expressed together they can form heteromeric channels with reduced channel properties compared to homomeric ones [22,27]. Although this has only been tested in ectopic expression systems it has been suggested that it might function as a mode of regulation in cells that endogenously express both proteins. Interestingly, the Panx1/Panx2 interaction only occurs with the Gly-0 and Gly-1 species of Panx1 [22] and it is unknown whether Panx2 glycosylation has any impact on the formation of these intermixed channels. Since previous studies have shown that Panx1 and Panx2 are often co-expressed in the same cells under normal conditions [15,18,27,28,29,30], and that their co-expression modulates important processes such as ischemia-induced neurodegeneration and brain damage in vivo [31], it is important to understand how these channels may be regulated by post-translational modifications (PTMs) such as N-glycosylation, and how this PTM may regulate their interaction. 

The present study aimed to validate the predicted N-linked-glycosylation site of Panx2 and determine its role in the regulation of the subcellular localization and intermixing of Panx2 with Panx1. We generated a Panx2 mutant protein completely devoid of N-glycosylation that, when overexpressed, exhibits a high level of intracellular aggregation with decreased traffic to the plasma membrane compared with wild-type Panx2. We found that N-glycosylation of Panx2 is not required for Panx1/Panx2 intermixing but facilitates Panx2 trafficking and localization at the plasma membrane when co-expressed with Panx1. The intracellular localization of un-glycosylated Panx2 reduces its co-localization with Panx1 at the cell surface and may impact their channel function in cells that co-express both glycoproteins, such as neurons. Collectively, we propose that N-glycosylation may be necessary for proper processing of Panx2 at the endoplasmic reticulum, regulating its intracellular distribution, but it is not required for interacting with Panx1.

## 2. Results

### 2.1. Characterization of the Panx2 N-Glycosylation Site at Asparagine 86

**Figure 1:**
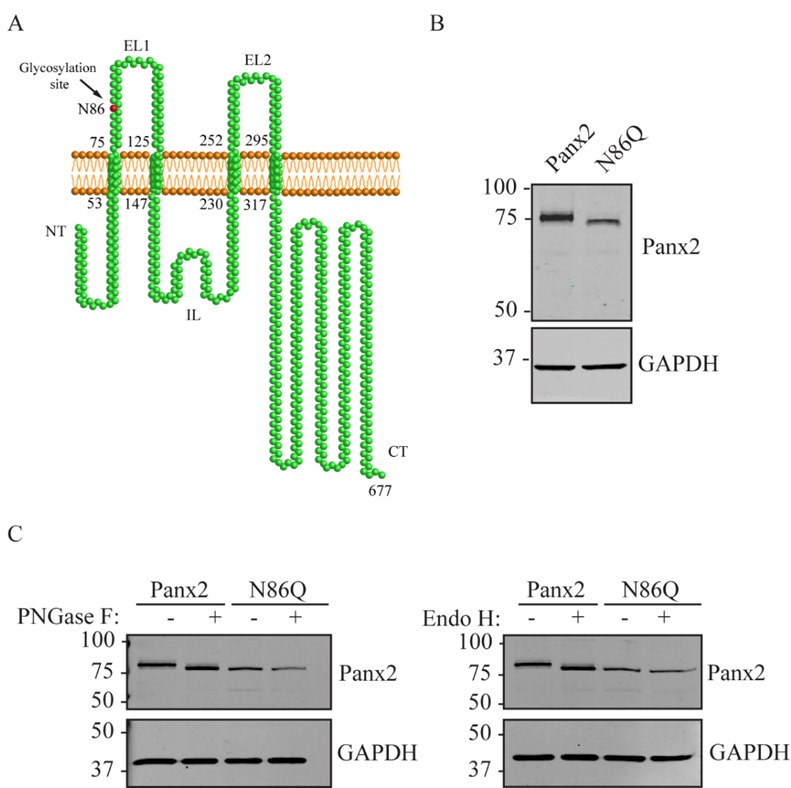
Asn86 is the N-glycosylation site of Panx2. (**A**) Based on sequence analysis, Panx2 (Uniprot ID: Q6IMP4-1) is predicted to contain four transmembrane domains, one intracellular (IL) and two extracellular loops (EL). The predicted N-glycosylation site is located at Asn86 in the first extracellular loop (EL1) (red residue). (**B**) Western blot (WB) comparing wildtype Panx2 and mutant N86Q, the latter shows a faster migrating band than the wildtype counterpart, indicative of decreased molecular weight. (**C**) Cell lysates of HEK293T transiently expressing Panx2 and N86Q mutant were subjected to enzymatic digestions with PNGase F and EndoH N-glycosidases. WB analysis confirmed that N86 is the only glycosylation site for Panx2 since only the wildtype protein exhibited a band shift after treatment with both glycosidases, and the de-glycosylated Panx2 band ran to the same position as the N86Q mutant. GAPDH was used as loading control. Molecular weights are noted in kDa.

Previous research has shown that ectopic expression of full-length mouse Panx2 presented N-glycosylated species with high-mannose modification [22]. Based on the analysis of the canonical sequence of Panx2 (UniProt ID: Q6IMP4-1), this modification might occur at asparagine 86 (Asn86 or N86) that is located in the first extracellular loop of Panx2 [20] (Figure 1A). To validate this prediction, we generated a full-length Panx2 mutant (Panx2^N86Q^, referred here as N86Q) with the substitution of the N86 with a glutamine (Gln, Q) that prevents the attachment of N-linked glycans to this specific site. As shown in Figure 1B, Western blotting (WB) of overexpressed constructs of N86Q and wild-type Panx2 (as glycosylated control) indicated that the electrophoretic band corresponding to N86Q mutant migrated faster than its wildtype counterpart, characteristic of a reduction in molecular weight.

To analyze whether N-glycosylation was prevented in the N86Q mutant, enzymatic de-glycosylation with endoglycosidase H (Endo H) and Peptide-N-Glycosidase F (PNGase F) were applied to the protein lysates and analyzed by WB (Figure 1C). Both de-glycosylation treatments did not cause any shift in migration of the N86Q band. Yet, wild-type Panx2 exhibited a small shift after treatment which relocated the migration of this band to the same position as that of the faster migrating N86Q mutant. Thus, the differences in the electrophoretic migration of the N86Q compared with the wild-type (WT) Panx2 can be explained by differences in molecular weight due to the absence of N-glycosylation. These findings validate N86 as the only N-glycosylation site present in Panx2 as was predicted by Penuela and others [22].

### 2.2. N86Q Forms Intracellular Aggregates

**Figure 2:**
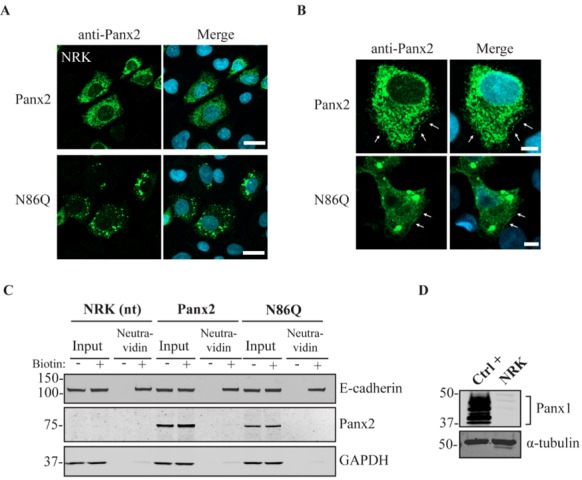
Panx2 has a predominantly intracellular localization, and its N-glycosylation-deficient mutant (N86Q) forms aggregates in NRK cells with low endogenous Panx1 protein expression. (**A**) Confocal micrographs of Panx2 and N86Q ectopically expressed in NRK cells, immunolabeled with anti-Panx2 antibody (green), revealed that Panx2 is predominantly localized intracellularly. The mutant N86Q is also localized intracellularly but appeared mostly as punctate aggregates. Nuclei (blue) were counterstained with Hoechst 33342. Scale bars = 20 μm. (**B**) Representative images of a small subpopulation of NRK cells expressing Panx2 or N86Q that showed minimal apparent localization at the cell surface (indicated with white arrows). Scale bars = 5 μm. (**C**) Western blot analysis of cell-surface-biotinylated proteins with EZ-Link™ Sulfo-NHS-SS-Biotin pulled down with NeutrAvidin^®^ beads showed very low detection of Panx2 but not the N86Q mutant at the cell surface of NRK cells. E-cadherin was used as a positive control of cell surface protein labeling and GAPDH was used as a negative control of intracellular proteins (no biotin internalization). Non-transfected NRKs (nt) were used as a negative control. (**D**) Western blot of protein lysate from NRK cells indicated that these cells have very low detectable levels of endogenous Panx1. Overexpressed Panx1 was used as positive control (Ctrl +) and endogenous α-tubulin was used as protein loading control. Molecular weights noted in kDa.

**Figure 3:**
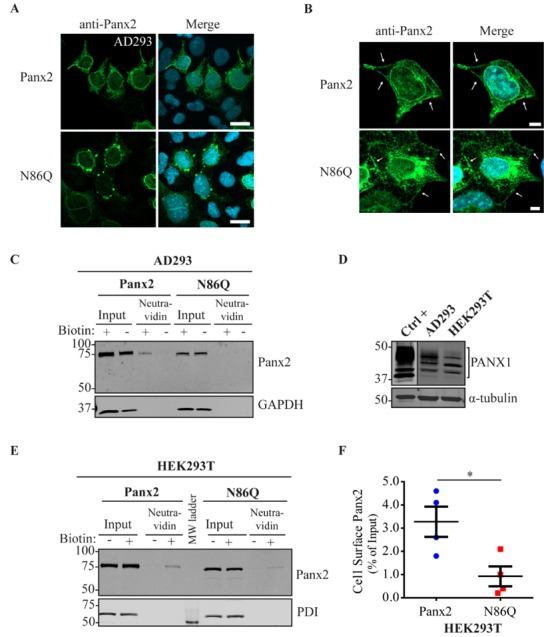
Analysis of Panx2 and N86Q localization in AD293 and HEK293T cells expressing endogenous PANX1. (**A**) Immunolabeling of Panx2 and N86Q mutant (green) showed that both localized mostly intracellularly, but the N86Q mutant aggregated intracellularly in AD293 cells. Scale bars = 20 μm. (**B**) A subpopulation of AD293 cells displayed Panx2 localization at the cell surface, less evident with the N86Q mutant (indicated with white arrows) Scale bars = 5 μm. Nuclei (blue). (**C**) Cell Surface Biotinylation Assays on AD293 cells showed a weak detection of the Panx2 wildtype but not N86Q mutant in surface-labeled fractions. GAPDH was used as a control for biotin internalization. (**D**) Immunoblots of AD293 and HEK293T cells confirmed that both cell lines express endogenous PANX1. Overexpressed human PANX1 served as positive control (Ctrl +) and endogenous α-tubulin was used as loading control. Line dividing upper panel of PANX1 WB indicates differences in exposure of the same blot to show a better detection of endogenous PANX1 compared to the overexpressed positive control. (**E**) Cell surface biotinylation experiments performed on HEK293T cells showed that overexpressed Panx2 and the mutant N86Q are detectable at the cell surface. Protein disulfide-isomerase (PDI) was used as a control for biotin internalization. (**F**) Densitometric analysis and quantification of cell surface biotinylation experiments performed in HEK293T cells revealed a significant reduction of N86Q cell surface detection compared to Panx2. Cell surface detection was calculated relative to the total protein in input lanes. Statistical significance was considered when *p* < 0.05 (* *p* = 0.0286, *N* = 4 independent experiments), Mann Whitney U test. Error bars denote mean ± S.E.M. Molecular weights are noted in kDa.

To determine whether glycosylation of Panx2 has any effect on its subcellular localization, we ectopically expressed Panx2 WT and N86Q in different reference cell lines and evaluated whether the intracellular distribution observed was dependent on the cell type as has been reported for Panx1 and Panx3 [32]. Because our group has demonstrated before [21] that Panx1 influences Panx2 trafficking, we selected Normal Rat Kidney cells (NRK), that in our experience have very low expression of endogenous Panx1 (Figure 2D), and “adherent” human embryonic kidney cells (AD293) that endogenously express the human ortholog PANX1 (Figure 3D). Likewise, these cell lines were selected as they are suitable transfection hosts with a relatively large cytoplasm which facilitates the visualization of the subcellular localization of proteins.

Confocal immunofluorescence imaging revealed an intracellular localization of ectopic Panx2 (Figure 2A and Figure 3A, top panel), with perinuclear distribution and spread in intracellular compartments. Interestingly, in both cell types assayed, the mutant N86Q localized intracellularly with large subpopulations forming punctate aggregates (Figure 2A and Figure 3A, bottom panel).

### 2.3. Limited Panx2 Localization at the Cell Surface Is Reliant on N-Glycosylation Status and the Level of Panx1

Despite the predominant intracellular localization, in a subpopulation of cells, Panx2 and the N86Q mutant (to a lesser extent) were apparent in limited regions of the cell surface (Figure 2B and Figure 3B, arrows). To corroborate these results, cell surface biotinylation assays followed by immunoblotting were conducted using a cell-impermeable biotinylation reagent (Sulfo-NHS-SS-Biotin). Cell-surface biotinylation experiments in NRKs (low endogenous Panx1, Figure 2D) showed a faint band of Panx2 and no detection of N86Q at the cell surface in the neutravidin pull-downs (Figure 2C). Also, AD293 cells (with a higher level of endogenous PANX1, Figure 3D) exhibited low Panx2 and no detectable N86Q mutant protein at the cell surface (Figure 3C). However, in subsequent experiments we also used human embryonic kidney (HEK293T) cells (Figure 3D) that have been used in previous studies [11,22], because of their increased transfection efficiency and enhanced protein expression due to the SV40 T-antigen [33]. After ectopic expression in HEK293T cells, we performed the same cell-surface biotinylation assays and noticed that Panx2 WT protein was detected (approximately 4% of the total Panx2 expression) at the cell surface (Figure 3E). Under these overexpression conditions, the N86Q mutant was also detected in the biotinylated-protein fractions (Figure 3E) but there was a significant (*p* = 0.0286, *N* = 4) reduction (to ~1% of its total amount) in the cell surface protein pool of the mutant (Figure 3F). Therefore, although the Panx2 cell membrane trafficking is reduced when Panx2 is not N-glycosylated at N86, its cell surface localization is not completely abrogated when overexpressed in HEK293T cells.

### 2.4. Panx2 and N86Q Aggregates Localize to the Endoplasmic Reticulum and Golgi Apparatus

**Figure 4:**
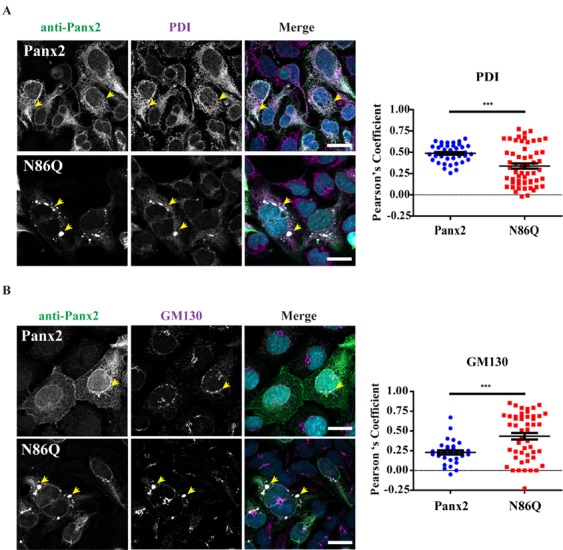
Panx2 and N86Q colocalize with markers of the endoplasmic reticulum and Golgi. Representative confocal micrographs of Panx2 and N86Q ectopically expressed in AD293 cells. Co-immunolabeling with anti-Panx2 antibody (green) and organelle markers (magenta): (**A**) PDI, endoplasmic reticulum (ER); (**B**) GM-130, cis-Golgi matrix. Panx2 has a perinuclear localization and is spread intracellularly in the cytoplasm partially colocalizing with markers of the endoplasmic reticulum and Golgi. N86Q aggregates also overlap with ER and Golgi markers and disrupt their distribution. Yellow arrowheads indicate representative regions of colocalization. Nuclei (blue) were counterstained with Hoechst 33342. Scale bars = 20 μm. Pearson Correlation Coefficients (right) were calculated for multiple regions of interest (ROI)s corresponding to double-labeled cells. Statistical significance was considered when *p* < 0.05 (*** *p* ≤ 0.0001, *N* = 3 independent experiments), using unpaired two-tailed *t* test with Welch’s correction. Error bars denote mean ± S.E.M.

**Figure 5:**
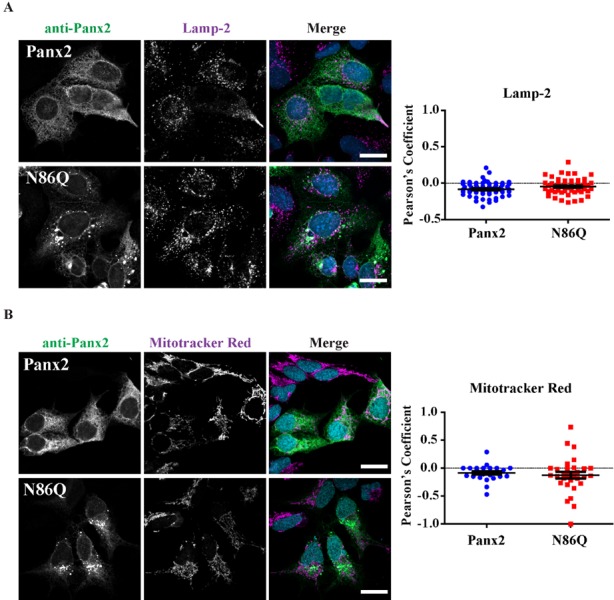
Panx2 and N86Q do not localize to late endosomes/lysosomes or mitochondria. Confocal micrographs of Panx2 and N86Q ectopically expressed in AD293 cells. Co-immunolabeling with anti-Panx2 antibody (green) and organelle markers (magenta): (**A**) Lamp-2, lysosomes and late endosomes, and (**B**) Mitotracker^®^ Red, mitochondria showed that neither Panx2 or N86Q mutant exhibited significant overlap with the markers. Nuclei (blue) were counterstained with Hoechst 33342. Nuclei (blue) were counterstained with Hoechst 33342. Scale bars = 20 μm. Pearson Correlation Coefficients (right) were calculated for multiples ROIs corresponding to double-labeled cells. There was no statistical significance (*p* > 0.05, *N* = 3, unpaired two-tailed *t* test with Welch’s correction) in the degree of colocalization between Panx2 and N86Q with the markers. Error bars denote mean ± S.E.M.

Because of the prominent intracellular localization of both Panx2 and the mutant N86Q, we were interested in determining the subcellular compartments to which these proteins could be trafficking. We transiently expressed these proteins in AD293 cells and used immunolabeling with different organelle markers to assess their intracellular location by confocal microscopy (Figure 4 and Figure 5).

As shown in Figure 4A, Panx2 immunolabeling exhibits a broad cytoplasmic distribution highly overlapping (Pearson’s Colocalization Coefficient (PCC)_Panx2-PDI_ = 0.49 ± 0.02; *n* = 41, *N* = 3) with the chaperone protein disulfide-isomerase (PDI), a known marker of the endoplasmic reticulum (ER). The mutant N86Q had significantly (*p* < 0.0001) less overlap (PCC_N86Q-PDI_ = 0.34 ± 0.03, *n* = 57, *N* = 3) with PDI although there was also a more punctate distribution of PDI that co-localized with N86Q. These observations suggest that N86Q is being confined to some punctate regions enriched in PDI, along with other subcellular organelles. We also observed an apparent alteration of the ER morphology when N86Q was expressed.

Quantitation of colocalization with cis-Golgi marker (GM130) (Figure 4B) showed a significantly (*p* = 0.0001) higher colocalization with the N86Q mutant (PCC_N86Q-GM130_ = 0.43 ± 0.04; *n* = 41, *N* = 3) than with Panx2 WT (PCC_Panx2-GM130_ = 0.23 ± 0.03; *n* = 30, *N* = 3). In this case, cells that overexpressed Panx2 WT exhibited changes in the distribution of GM130 compared to un-transfected ones (in the same field of view, Figure 4B). These changes were more pronounced in AD293 cells overexpressing the mutant N86Q, in which not only the cis-Golgi morphology changed, but GM130 also appeared to accumulate within N86Q aggregates. 

On the other hand, lysosome-associated membrane protein 2 (Lamp-2) and the fixable and cell permeant Mitotracker™ Red CMXRos were assayed to label lysosomes/late endosomes, and active mitochondria, respectively. We did not observe colocalization with Lamp-2 (PCC_Panx2-Lamp-2_ = −0.08 ± 0.01; *n* = 59, *N* = 3; PCC_N86Q-Lamp-2_ = −0.05 ± 0.02; *n* = 49, *N* = 3) or Mitotracker (PCC_Panx2-Mitotracker_ = −0.08 ± 0.03; *n* = 25, *N* = 3; PCC_N86Q-Mitotracker_ = −0.13 ± 0.06; *n* = 31, *N* = 3), and there was no difference in the distribution of both markers upon overexpression of Panx2 WT or N86Q.

### 2.5. Panx2 N-Glycosylation Is Not Required for the Interaction with Panx1

**Figure 6:**
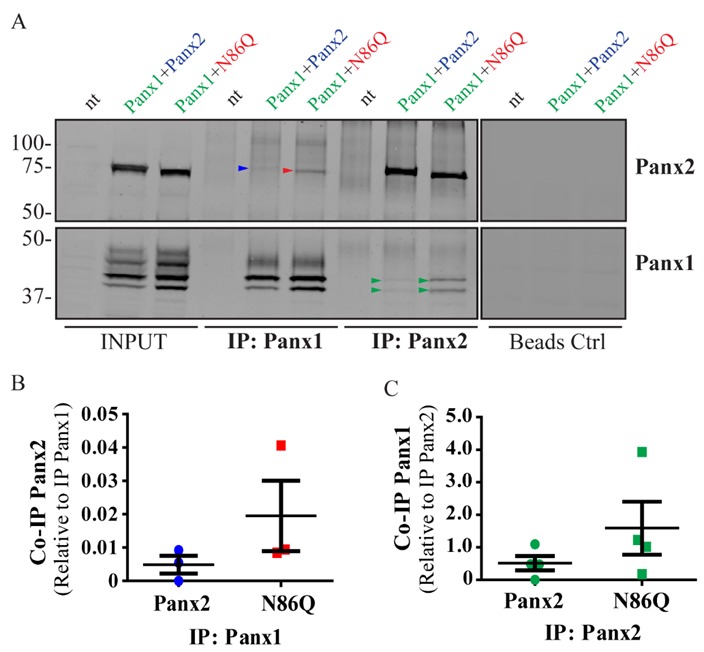
Panx2 glycosylation is not required for the interaction with Panx1 by immunoprecipitation. (**A**) Reciprocal co-immunoprecipitation (co-IP) experiments showed that both Panx2 and N86Q co-IP in a complex with overexpressed Panx1 in HEK293T cells. Colored arrowheads denote bands of co-IP proteins detected in WB. (**B**,**C**) Quantitative analysis (see Materials and Methods Section 4.8) of co-IP shows that the interaction of N86Q with Panx1 is not significantly different (*p* > 0.05, *N* = 4) than with Panx2, and in both cases the complexes only involved the lower glycosylated species of Panx1 (Gly-0 and Gly-1). Beads Ctrl denote control IPs done in parallel without antibodies. Protein sizes in kDa.

Due to our previous report [22] in which we showed that glycosylation regulates intermixing of pannexins and that Panx2 interacts only with the core (non-glycosylated, Gly-0) and high-mannose species (Gly-1) of Panx1, we were interested in determining what would be the outcome with the un-glycosylated species of Panx2. For these experiments, Panx1 was ectopically co-expressed with either Panx2 or non-glycosylated N86Q mutant in HEK293T cells. Using co-immunoprecipitation assays (co-IP, Figure 6A), we observed that both Panx2 and N86Q can co-IP in a complex with Panx1 and occasionally, although not statistically significant (*p* > 0.05, *N* = 4) (Figure 6B,C), N86Q pulled down more Panx1 than the WT Panx2. In addition, consistently with what was reported before by Penuela et al. (2009), we noticed that only Gly-0 and Gly-1 Panx1 species interacted with both variants of Panx2.

**Figure 7:**
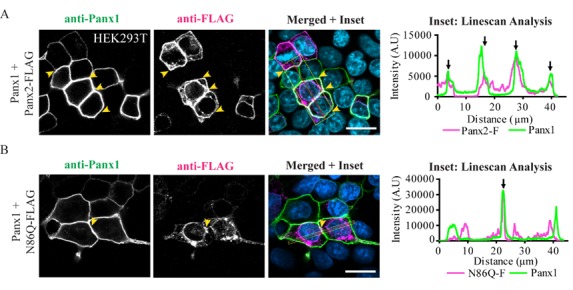
N-glycosylation may enhance plasma membrane localization of Panx2 when co-expressed with Panx1. Confocal micrographs of (**A**) Panx2-FLAG or (**B**) N86Q-FLAG (magenta) ectopically co-expressed with mouse Panx1 (green) in HEK293T cells. 72 h post-transfection Panx2-FLAG partially colocalized with Panx1 at the cell membrane (see black arrows in Linescan, **panel A**) with a subpopulation still in intracellular compartments. N86Q-FLAG formed intracellular aggregates and showed limited colocalization with Panx1 at the plasma membrane (see black arrows in Linescan, **panel B**). Yellow arrowheads denote regions of colocalization of Panx1 and FLAG labeling also depicted with black arrows in the corresponding linescans. Insets: Linescans showing the overlapping (black arrows) between fluorescence peaks to denote colocalization. Nuclei (blue, Hoechst 33342). Scale bars = 20 µm.

Due to the lack of available antibodies from different species to perform double immunolabeling of both pannexins, we used C-terminal FLAG-tagged Panx2 and N86Q that were co-expressed with Panx1 in HEK293T cells. Confocal imaging of Panx2-FLAG or N86Q-FLAG co-expressed with Panx1 (Figure 7A) showed that Panx2-FLAG exhibits both an intracellular and cell surface localization overlap with Panx1 (see Linescan analysis, Figure 7A). N86Q-FLAG formed mostly intracellular aggregates like its untagged counterpart (Figure 3A). A small subpopulation of N86Q-FLAG could still be seen at the cell surface colocalizing with Panx1 (see Linescan analysis, Figure 7B), but to a lesser degree than Panx2-FLAG. Taken together, these findings suggest that the glycosylation of Panx2 is not required for the interaction of Panx1/Panx2 but can determine the differential localization of glycosylated and un-glycosylated species.

## 3. Discussion

Pannexins are a family of channel proteins implicated in important physiological and pathological functions and most of the current research has been conducted to analyze their level of expression and distribution within mammalian tissues and their role in diverse diseases [34]. However, there is still a need to understand the biophysical properties of these channels and the different ways of regulation that prevent the detrimental effects of their exacerbated channel activity. Pannexins are N-glycosylated and as integral membrane proteins, this modification seems to be essential in regulating their trafficking, as was demonstrated formerly for Panx1 and Panx3 [11,22]. Unlike the other pannexins, Panx2 is modified only to a high-mannose glycosylation (Gly-1) which is known to be an early post-translational modification occurring in the ER lumen. For many other glycoproteins, this step is generally followed by further oligosaccharide editing in the Golgi (complex glycosylation). To date, there is no evidence showing further processing of Panx2 in Golgi and only studies in Panx1 and Panx3 showed that trafficking of these two pannexins to the plasma membrane is mediated by Sar1-dependent COPII vesicles [35] with N-glycosylation affecting their final delivery [11,36,37]. This suggests that N-glycosylation regulates the route of pannexin trafficking and modifies their localization and channel formation in immortalized culture cells. A previous report of Panx2 localization pointed to a predominantly intracellular distribution that can also be modified by other PTMs like palmytoilation, which can determine its subcellular localization in neurons [17].

Panx2 seems to be mostly intracellularly localized, but in some instances, it can also translocate to the plasma membrane [14,17]. However, the exact site of N-glycosylation and whether this modification affects Panx2 trafficking was unknown. In this study, we sought to determine the N-glycosylation site by generating a glycosylation-deficient mutant (N86Q) based on the predicted N-linked glycosylation site reported by Penuela et al. [22]. For our experiments we transfected constructs encoding mouse Panx2 and an N86Q mutant into HEK293T cells. Our results showed that N86Q substitution generated a Panx2 mutant with a faster-migrating electrophoretic band compared to Panx2 WT, that does not shift after specific N-glycosidase digestion with endoglycosidases, thus confirming that N86 is the only N-glycosylation site for Panx2. In the other cell lines assayed (NRK and AD293) we also observed that overexpression of the same Panx2 WT and N86Q constructs exhibited the same electrophoretic properties seen with HEK293T (Figure 2C and Figure 3C). 

Independently of the cell-type used for ectopic expression, the N86Q mutant appeared to form punctate aggregates that were localized to intracellular compartments along with the ER-chaperone PDI, and the cis-Golgi matrix marker GM130. Compared to the N-glycosylation-deficient mutants of Panx1 and Panx3 [11], un-glycosylated Panx2 exhibited an exacerbated abnormal intracellular aggregation. This raises the possibility that the lack of N-glycosylation may have affected proper protein folding of Panx2, which is the largest member of the pannexin family. We cannot rule out that the intracellular accumulation of N86Q could be an artifact of overexpression, that concomitant with the lack of glycosylation of the Panx2 mutant, might have induced misfolding and ER-stress [38].

A previous study in murine postnatal hippocampal neural progenitor cells (NPC)s [17] showed that treatment with glycosidases had no effect on endogenous Panx2 electrophoretic mobility, suggesting that they were un-glycosylated. That study relied on antibody detection of endogenous Panx2, and the protein bands identified were of lower molecular weight than the predicted full-length Panx2 used in this study (677 aa, [39]). Further studies are needed to determine first, if the expression of certain un-glycosylated endogenous isoforms of Panx2 is cell-type specific, and second, whether glycosylation has measurable effects on the Panx2 cellular function. To date, there are no reports of mutations in the *Panx2* gene, but our results would predict significant changes in Panx2 behavior if its glycosylation is affected. It is also possible that the un-glycosylated form of Panx2 may be preferentially expressed in some cells and tissues determining the primary function of the Panx2 channel and its subcellular localization.

In our study, we detected large intracellular subpopulations of Panx2 likely localized in the ER, and partially colocalized with cis-Golgi marker. Interestingly, we found that overexpression of the N86Q mutant changed the distribution of PDI and GM130 compared to Panx2 WT, suggesting that the formation of aggregates may disrupt the morphology of ER and cis-Golgi. GM-130 is a peripheral membrane protein in *cis*-Golgi matrix that is important for maintenance of Golgi structure [40], and the regulation of ER-to-Golgi transport of proteins and glycosylation [41]. It is possible that the aggregation of N86Q may interfere with the mutant protein transport from the ER causing accumulation of GM130. 

As reviewed by Boyce et al. [42], pannexins contain putative recognition sequences for endocytic and endo-lysosomal targeting which could account for the control of pannexin trafficking. Interestingly, recently published work by Boassa et al., described the localization of a recombinant Panx2 fused to mini-SOG tag that was transiently expressed in HeLa cells [26]. These authors used correlated light and electron microscopy imaging to detect Panx2 localization at cytoplasmic protrusions. Also, immuno-colocalization in HEK293T cells using assorted vesicular markers displayed Panx2 WT (untagged) localized to early or recycling endosomes rather than ER. Although, they mentioned in the manuscript that when the ER marker calnexin was used they detected colocalization with overexpressed Panx2 in HEK293T cells. Our findings are consistent with these reports [24], since we found Panx2 primarily in the ER (Figure 4A). However, we did not find conclusive evidence of Panx2 in endo-lysosome compartments (Figure 5A) as others have reported [25,26]. Based on our results, it is possible that Panx2 may have an intracellular channel function in the ER, similar to the proposed calcium-leak channels formed by Panx1 and Panx3 [6].

In some instances, Panx2 distribution exhibited limited cell surface localization that was more apparent when higher endogenous or ectopic Panx1 protein was expressed in the studied cell lines. This is consistent with our previous observation of increased Panx2 at the cell surface when co-expressed with Panx1 under overexpression conditions [22]. Here, we used three different cells lines with varying levels of endogenous Panx1 and a different capacity of protein production. We noticed that in cells with low endogenous Panx1 (e.g., NRKs), there was barely any Panx2 at the cell surface based on immunolabeling and cell surface biotinylation pull-downs. In the case of AD293 and HEK293T cells, it was possible to detect low levels of overexpressed Panx2 at the cell surface, while the mutant Panx2 (N86Q) had a detectable but significantly decreased presence only at the cell membrane of HEK293T cells. This result could be attributed to the increased protein expression in HEK293T cells that contains the SV40 T-antigen and have high transfection efficiency. This feature might have allowed the overexpressed Panx2 to bypass mechanisms of protein quality control resulting in more Panx2 trafficking to the plasma membrane [43].

Further research is needed to evaluate endogenous Panx2 in terms of N-glycosylation and subcellular localization of its isoforms, and to examine if Panx2 can form channels at the plasma membrane under physiological conditions. To date, limited studies have attempted to evaluate the Panx2 channel function [14,15] and several factors make it difficult to test Panx2 channel activity, such as its intracellular localization, the lack of evidence of in vivo functional channel formation [27] and the unknown mechanisms of activation [14].

Work done by Bruzzone et al. [15], showed that Panx1 and Panx2 were abundantly expressed in the CNS, and co-injection of both pannexin RNAs in paired *Xenopus* oocytes resulted in the formation of heteromeric channels with functional characteristics different from those formed by Panx1 monomers but with similar pharmacological sensitivity [27]. Ambrosi et al. [14] suggested that Panx1/Panx2 heteromeric channels tend to be unstable and they attributed that to differences in monomers size and oligomeric symmetry between these two pannexins. We have previously shown that Panx1 and Panx2 do form a complex as determined by co-IP experiments. Interestingly, when both pannexins are co-expressed, the level of interaction between Panx2 and glycosylated-species of Panx1 is dependent of the glycosylation of the latter [22]. Here, we showed in vitro, that the Panx2 glycosylation-deficient mutant can readily form complexes with Panx1, thus Panx2 glycosylation is not required for intermixing of the two pannexins. In fact, although it was not statistically significant, N86Q seemed to pull-down Panx1 more efficiently than the WT Panx2. However, in confocal images the N86Q aggregates did not show higher overlap with Panx1-immunolabeling than Panx2 WT. Consistent with our previous report [22], complex N-glycosylation of Panx1 hinders their interaction, since both Panx2 and N86Q interacted only with the Gly-0 and Gly-1 forms of Panx1. Taken together, these results suggest that in an ectopic expression system, glycosylation of Panx2 is not required for Panx1/Panx2 intermixing, but it does help with the transport of Panx2 to the cell surface, which is also increased by the presence of Panx1. 

Finally, we propose that N-glycosylation of pannexins is an important post-translational modification that partially regulates their subcellular localization. Whether N-glycosylation represents a post-translational mechanism that regulates trafficking and Panx1/Panx2 interactions in vivo would be important questions to address in future studies. In cells where both pannexins are co-expressed, glycosylation may act as a form of regulation defining whether these channels will serve as intracellular or plasma membrane channels with different physiological and pathological functions. 

## 4. Materials and Methods

### 4.1. Cell Lines, Constructs and Transient Transfections

Media, supplements and reagents were obtained from GIBCO^®^ and Invitrogen™ (Carlsbad, CA, USA). Normal rat kidney (NRK) (ATCC^®^ CRL-6509™) and human embryonic kidney cells (HEK293T) (ATCC^®^ CRL-3216™) were obtained from ATCC (Manassas, VA, USA). Adherent HEK293 cells (AD293, Cat# 240085) were obtained from Agilent Technologies, Inc. (Santa Clara, CA, USA). Cell cultures were grown in high-glucose DMEM supplemented with 10% fetal bovine serum (FBS), 100 U/mL penicillin, 100 µg/mL streptomycin and 2mM L-Glutamine. At ~50% of confluency, cells were transfected adding Lipofectamine 3000 (Invitrogen™) following manufacturer directions. 2 µg of pcDNA3.1 (Invitrogen™) plasmids encoding mouse Panx2 [22] or Panx2^N86Q^ or their respective FLAG-tagged versions were used for transfections in 35 mm culture plates. After 48 h for single transfections and 72 h for co-transfections, proteins were extracted, and expression levels were determined by Western blot. For co-transfections experiments with mPanx1 plasmid [11], levels were reduced to 0.5 µg of DNA.

### 4.2. Mutagenesis and Cloning of New FLAG-Tagged Panx2 Constructs

As described previously [11], Panx2 has a predicted N-glycosylation consensus site located at asparagine (N) 86 on the first extracellular loop. Site-directed mutagenesis service (NorClone Biotech Labs, London, ON, Canada) was used to generate a new expression Panx2 construct encoding a replacement of asparagine by glutamine at position 86, referred to as Panx2 N86Q. FLAG-tagged Panx2 was obtained by inserting a single FLAG sequence with In-Fusion HD Cloning Kit (Clontech Laboratories, Inc., Takara Bio, Inc., Mountain View, CA, USA) at the end of the coding region of the Panx2 C-termini. Primers used for FLAG insertion were Forward: 5′-GTTTAAACTTAAGCTTCATGCACCACCTCCTGGAG-3′ and Reverse: 5′-GCCCTCTAGACTCGAGCTCACTTGTCATCGTCGTCCTTGTAATCAAACTCCACAGTACT-3′. All the constructs were verified by sequencing.

### 4.3. Protein Extractions and Western Blots

For co-immunoprecipitation (Co-IP) assays, cell lysates were obtained using a Triton-based extraction buffer (IP buffer) (1% Triton X-100, 150 mM NaCl, 10 mM Tris, 1 mM EDTA, 1 mM EGTA, 0.5% NP-40). The rest of the protein extractions were performed with SDS-based buffer (RIPA buffer) (0.1% SDS, 50 mM Tris-HCl, pH 8.0, 150 mM NaCl, 1% NP-40 and 0.5% Sodium Deoxycholate). In each case, lysis buffers were complemented with a final concentration of 1 mM NaF, 1 mM Na_3_VO_4_, and one tablet of cOmplete™-mini, EDTA-free Protease Inhibitor Cocktail (Roche, Mannheim, Germany). Total protein concentrations were quantitated with Pierce™ BCA Protein Assay Kit (Thermo Scientific, Rockford, IL, USA). For Western Blots, 50 µg of total protein were resolved in 8% SDS-PAGE and transferred onto nitrocellulose membranes using an iBlot™ Blotting System (Invitrogen, Carlsbad, CA, USA). Membranes were blocked with 3% bovine serum albumin (BSA, Burlington, ON, Canada) and 0.05% Tween 20-Phosphate Buffer Saline (T-PBS) for 45 min at room temperature and probed overnight with a 1:1000 dilution of the rabbit affinity-purified antibodies anti-Panx2-CT-523 [22]. Mouse monoclonal anti-FLAG^®^ M2 (Sigma, St. Louis, MO, USA, Cat# F3165), monoclonal mouse anti-GAPDH (Millipore, Burlington, MA, USA, Cat# MAB374, RRID: AB_2107445), and anti-α-Tubulin (Millipore-Sigma Cat# 05-829, clone DM1A) antibodies were used at 1:2000, 1:1000 and 1:5000 dilutions, respectively. For detection, IRDye-800 and -680RD (Life Technologies™, Carlsbad, CA, USA) were used as secondary antibodies at 1:10,000 dilution and the membranes were scanned on a Li-Cor Odyssey infrared system (Li-Cor, Lincoln, NL, USA). In most cases, GAPDH was used as a loading control.

### 4.4. Immunofluorescence, Confocal Imaging, Linescans and Colocalization Analysis

Cells were grown on coverslips at ~70% of confluency and were transfected as described previously in Section 4.1. After 48 h of transfection, coverslips were washed with D-PBS (Gibco^®^) and fixed with ice-cold 80% methanol and 20% acetone for 15 min at 4 °C. Coverslips were blocked with 2% BSA-PBS for 1h and primary antibodies were used diluted in blocking buffer as follows: polyclonal anti-Panx2-CT (2 mg/mL, 1:100 dilution), polyclonal anti-Panx1-CT (1 mg/mL, 1:100 dilution), anti-PDI monoclonal antibody (1 mg/mL, 1:400 dilution) (1D3, Enzo^®^ Life Sciences, Burlington, ON, Canada, ADI-SPA-891-D), cis-Golgi marker anti-GM130 (1 mg/mL, 1:300) (Abcam, Toronto, ON, Canada, Prod#: ab169276), anti-FLAG^®^ M2 (1 mg/mL, 1:500) (F3165, Sigma, St. Louis, MO, USA), mouse monoclonal anti-Lamp-2 (1 mg/mL, 1:300) (DHSB, clone H4B4). Coverslips were incubated with primary antibodies for 1 h at room temperature, then washed with PBS and incubated with the secondary antibodies: Alexa Fluor 488 goat anti-rabbit IgG (2 mg/mL, 1:700) or goat anti-mouse antibody Alexa Fluor 647 (2 mg/mL, 1:400) (Life Technologies), that were selected to avoid bleed-through between dyes. Mitochondrial labeling was performed by using MitoTracker™ Red CMXRos (M7512, Thermofisher, Life Technologies, Eugene, OR, USA) as per manufacturer directions, and cells were fixed with freshly prepared paraformaldehyde (4%) and then permeabilized with 0.1% Triton X-100 before the blocking step. Coverslips were rinsed with PBS followed by water once and counterstained with Hoechst 33342 (H3570, Life Technologies™, Eugene, OR, USA) (1:1000, in water) for 5 min to stain nuclei and then were mounted with the custom-made Airvol mounting medium. Imaging was performed with an LSM 800 Confocal Microscope (Carl Zeiss, Oberkochen, Germany) using a Plan-Apochromat 63x/1.40 Oil DIC objective (Carl Zeiss, Oberkochen, Germany). Image acquisition for colocalization was performed with sequential laser scanning and with the multitracking feature of the Zeiss software with settings to avoid wrong excitation-crosstalk and bleed-through of the channels. Colocalization was quantitated with the colocalization plug-in of the Zeiss software (ZEN, version 2.3, blue edition). Regions of interest (ROI) were drawn in dual-labeled cells selecting individual cells expressing Panx2 or the mutant and co-stained with organelle markers. Controls of single-labeled cells were used to determine thresholds of intensities for each single channel and a manual thresholding was used to determine the region of pixels colocalized in the intensities scatterplots. Pearson correlation coefficient was determined for each cell as a measure of colocalization and was expressed as means ± S.E.M., representative of at least three independent transfections. Linescans using Zeiss software tool were used to detect overlaps of fluorescence peaks in cells co-transfected with Panx1. 

### 4.5. Cell Surface Biotinylation Assays

Cell surface biotinylation assays were performed as described in Reference [22]. Briefly, cells were grown in 60 mm plates and used for biotinylation 72 h following transfection with Panx2 or N86Q constructs. After, culture media was aspirated, the cell monolayer was rinsed twice with ice-cold D-PBS supplemented with Ca^2+^ and Mg^2+^ (Gibco^®^). Then, cells were incubated only with D-PBS (non-labeling as a negative control) or with a solution of 1.5 mg/mL EZ-Link™ Sulfo-NHS-SS-Biotin (Thermo Scientific, Rockford, IL, USA) in D-PBS for 30 min on ice and covered from light. Plates were washed once again with D-PBS and then incubated with 100 mM glycine dissolved in D-PBS for 30 min to quench the remaining labeling biotin washed once more with D-PBS. Lysates were prepared using RIPA buffer as described before. For pull-down of cell surface biotinylated proteins, 250 µg of total protein was incubated overnight with 50% slurry of 50 µL NeutrAvidin agarose beads (Thermo Scientific, Rockford, IL, USA). Samples from lysates and initial flow-through wash were collected, the beads were spun down (at 500× *g*, 4 °C) and then washed three times with RIPA buffer. The samples and the beads were then mixed with 2X Laemmli buffer and 10% β-mercaptoethanol and placed at 95 °C in heat block for 5 min. 50 µg of total protein from lysates and the beads supernatant were resolved in parallel with 8% SDS-PAGE gel and then transferred to nitrocellulose membranes as previously described. PDI or GAPDH were used as controls of non-specific biotinylation of intracellular/cytoplasmic proteins and E-cadherin was used as positive control of cell surface protein. 

### 4.6. Co-Immunoprecipitation (Co-IP) Assays

Co-immunoprecipitation (Co-IP) of protein complexes was performed at 4 °C by incubating overnight 1 mg of total protein from pre-cleared (with Protein A/G beads alone) lysates with 5 µg/mL of rabbit polyclonal anti-Panx2-CT or anti-Panx1-CT affinity-purified antibody crosslinked to Pierce Protein A/G-Agarose beads (Thermo Scientific); the same amount of beads (~30 µL) were used for IP in each case. Control experiments to evaluate unspecific binding to the beads were performed in parallel using beads with no antibody. To remove un-bound proteins, four washes with 500 µL of ice-cold IP buffer were performed. Then, beads were dried by aspiration and re-suspended in 2X Laemmli buffer (10% (*v*:*v*) β-mercaptoethanol), boiled for 5 min, spun down and the supernatants (IP samples) were used for WB. For WB analysis, 50 µg of protein of each lysate was loaded into the INPUT lanes and ran along with the IP samples. The intensities of the bands in each lane were obtained by densitometry and were used for quantitation.

### 4.7. De-Glycosylation Assays

Lysates from HEK293T cells ectopically expressing mouse Panx2 and the N86Q were used for validation of N-glycosylation site of the mouse Panx2 construct. Enzymatic de-glycosylation with Peptide-N-glycosidase F (PNGase F) and Endoglycosidase H (EndoH) were used to detect the presence of all complex forms of N-glycosylation and high-mannose modification, respectively. PNGase F (Roche, Indianapolis, IN, USA) and EndoH (New England Biolabs Ipswich, MA, USA) digestions were performed according to their manufacturer’s instructions. Briefly, at least 35 µg of total protein was denatured at 100 °C for 5 min in denaturing buffer (0.1% (*v*/*v*) SDS, 0.05 M 2-mercaptoethanol, 50 mM phosphate buffer, pH 7.5) and subsequently incubated for 1h at 37 °C with 10 units of the PNGase F, 0.7% (*v*/*v*) of Triton X-100 or 2500 U of Endo H in supplier’s digestion buffer. In the parallel control, samples were assayed without endoglycosidases. Protein samples were separated on an 8% SDS-polyacrylamide gel electrophoresis gel (PAGE) and transferred to nitrocellulose membranes for WB.

### 4.8. Densitometric Analysis of Western Blots

Densitometry analysis was performed in the Odyssey Application Software Version 3.0.16 (LI-COR Biosciences, Lincoln, NL, USA) as follows: For cell surface biotinylation experiments, the fraction of biotinylated-protein detected at the cell surface was calculated using the integrated intensity (I.I.) of protein bands detected in the Neutravidin lanes divided by the I.I. of protein bands detected in their corresponding input-lysate lanes. For Co-IP experiments, quantitative analysis was performed by calculating the ratio of the I.I detected for Co-IP protein divided by the I.I of the IP-target protein. Quantitation results were expressed as means ± S.E.M. representative of at least three independent experiments.

### 4.9. Statistics

Statistical analysis was performed using the statistical package of GraphPad Prism^®^ Ver. 5.03 (GraphPad Software, Inc., San Diego, CA, USA). Cell surface biotinylation data were analyzed with non-parametric Mann-Whitney U test for unpaired data. Data derived from quantitation of Co-IP assays and colocalization were analyzed with a two-tailed unpaired t test with Welch’s correction. A probability of *p* < 0.05 was considered as statistically significant.

## Abbreviations

AD293	Adherent human embryonic kidney cells
ATP	Adenosine triphosphate nucleotide
CNS	Central nervous system
Co-IP	Co-immunoprecipitation
Endo H	Endoglycosidase H
ER	Endoplasmic reticulum
GAPDH	Glyceraldehyde 3-phosphate dehydrogenase
GM130	Golgi matrix protein 130 kDa
HEK293T	Human embryonic kidney cells with SV40 T-antigen
IP	Immunoprecipitation
LAMP2	Lysosome-associated membrane glycoprotein 2
NPC	Neural progenitor cells
NRK	Normal rat kidney cells
PDI	Protein disulfide isomerase
PNGase F	Peptide-N-glycosidase F
PTM	Posttranslational modification
SV40	Simian vacuolating virus 40
